# Integration of community-engaged research technology and methods to guide tobacco regulatory science in rural communities

**DOI:** 10.3389/fpubh.2026.1867960

**Published:** 2026-07-24

**Authors:** Melissa H. Abadi, Stephen R. Shamblen, Mary Kay Rayens, Kirsten T. Thompson, Amy Wildman, Kathy Rademacher, William Wieczorek, Raphael Nishimura, Shyanika W. Rose, Mikhail N. Koffarnus, Seth Himelhoch, Ellen J. Hahn

**Affiliations:** 1 Pacific Institute for Research and Evaluation, Louisville Center, Louisville, KY, United States; 2 University of Kentucky, College of Nursing, Lexington, KY, United States; 3 Pacific Institute for Research and Evaluation, National Capital Region, Beltsville, MD, United States; 4 University of Michigan, Survey Research Center, Institute for Social Research, Ann Arbor, MI, United States; 5 University of Kentucky, College of Medicine, Lexington, KY, United States; 6 Division of the Biological Sciences, University of Chicago, Chicago, IL, United States

**Keywords:** community-engaged research, methods, rural, technology, tobacco, tobacco regulatory science

## Abstract

**Objective:**

This study aims to build a rural Appalachian cohort to examine how US Food and Drug Administration (FDA) Center for Tobacco Products (CTP) regulatory policies influence tobacco and nicotine product use across levels of rurality.

**Methods:**

The AppalTRUST (Appalachian Tobacco Regulatory Science Team) Cohort integrates four inter-connected Community-engaged Research + Technology (CEnRT) elements to support recruitment and retention: (1) building trust and cultural relevance; (2) addressing barriers to participation in research; (3) focusing on ethics and confidentiality; and (4) fostering ongoing and consistent collaboration between the scientific and field teams. The study uses a dual sampling design to recruit 1,000 quota-based non-probability sample participants and 1,000 address-based probability sample participants. Eligible adults (ages 18+) are recruited from two Appalachian Kentucky catchments spanning rural and peri-urban counties. A local field team combines culturally relevant engagement strategies with technology-enabled procedures, including virtual consent and participant tracking, to foster trust and relevance with an underserved population.

**Anticipated results:**

Recruitment is ongoing. Feasibility indicators include enrollment of 985 non-probability sample participants and 209 probability sample participants, a 16% direct-mail response rate, and a 62% participant preference for text-based consent and survey reminders. Expected outcomes include guidance for applying CEnRT approaches in underserved populations, characterization of tobacco and nicotine use trends across the rural–peri-urban continuum, and description of marketing exposure and consumer choice among subgroups of young adults and daily users in Appalachia. Findings will inform FDA CTP regulatory decision-making in rural Appalachian communities.

**Discussion:**

This integrated CEnRT approach shows promise for recruiting and retaining rural Appalachian adults by centering community voice, trust, confidentiality, and practical engagement strategies.

## 1 Introduction

### 1.1 Tobacco use and related disparities in Appalachia and Kentucky

Rural populations, including those in Appalachia, use tobacco and nicotine products at almost double the national rate (1). As of 2018–2019, KY had a tobacco product use prevalence among adults that was nearly 10 points above the national average (24.8 vs. 15.4%) (2). And, more recently, the estimated use rate of any tobacco product was higher in rural (32.4%) vs. urban (26.4%) locations in the state. (3) Persons living in rural Appalachia may experience multi-generational tobacco use and pro-tobacco norms due, in part, to higher exposure to tobacco industry advertising, point-of sale marketing of emerging products, and price promotion strategies, (4) as well as weak tobacco control (5) and related policies (e.g., fewer smoke-free laws in public places, and lower taxes) (6). These disparities in rural Appalachia contribute to high rates of tobacco-related disease, which are significantly higher compared to other areas of Appalachia (7).

Despite high tobacco use rates and related disparities, Appalachian populations have been largely overlooked in rigorous public health and tobacco research due to the challenges of engaging and surveying them through traditional recruitment and retention methods (8). Such challenges include (a) lack of access to households and community members due to remote and hard to reach residences (9–11); (b) lack of or limited broadband internet and digital communications (8, 12–14); (c) low reading (8) and technology literacy (15); and (d) mistrust of research and outsiders (16–18). Although these challenges are difficult and costly to address, failing to do so leaves rural populations underrepresented or misrepresented in public health research, which may lead to policies, programs, strategies, and services that are not well aligned with their communities.

### 1.2 Tobacco regulatory science (TRS)

Federal tobacco regulatory policies enacted by the US Food and Drug Administration (FDA) are critical to curbing the tobacco epidemic in rural Appalachian communities. Rural communities have often been left behind in local tobacco control policies, with lower tobacco taxes and lower likelihood of living in an area with comprehensive smoke-free policy coverage. Therefore, national regulatory policies have great potential to reduce rural disparities. However, to best do so, it is critical to determine which tobacco regulations are beneficial or harmful for rural Appalachian populations and what impact they might have. The US FDA implements policies to regulate tobacco manufacturing, marketing, and distribution of all tobacco products. Regulatory policies include determining which new products are allowed to come onto the market, product standards (e.g., menthol bans or lowering nicotine content in cigarettes), and what types of marketing claims products can make, and they support relevant and foundational research through a variety of mechanisms including through tobacco centers of regulatory science (TCORS). This paper describes the sampling design and research methods our TCORS, AppalTRUST (Appalachian Tobacco Regulatory Science Team), used to build an Appalachian Cohort, engage rural residents in tobacco regulatory research, inform FDA regulations, and reduce rural tobacco use disparities.

### 1.3 Using community-engaged research (CEnR) methods to recruit and retain the AppalTRUST cohort

Addressing the regulatory science gap in rural Appalachia requires methodologies that can authentically reach and retain underserved populations who have historically been excluded from tobacco research. Community-engaged research (CEnR) offers such an approach. Although rarely used in tobacco regulatory science, CEnR is an approach in which researchers meaningfully engage and collaborate with community members, organizations, influencers, and stakeholders to co-design and co-implement studies that are aligned with the culture, geography, experiences, and preferences of the population (19, 20). Communities are not merely research settings, but they are also where authentic research relationships are built, and community members are active contributors and experts who inform the research methods, help interpret findings, and help report back the data to their communities (21). In this way, trust is built between the researcher and community, and there are shared goals for success.

To recruit and maintain the AppalTRUST Cohort, we prioritize authentic and consistent collaboration with the communities being surveyed and integrate technological methods to ensure that best practices for community-engaged research are integrated with the most effective and up-to-date methods for survey research. As such, we have developed a CEnRT (community-engaged research + technology) framework. Importantly, these technological integrations also provide ongoing and reliable support to our local field team to ensure efficiency and effectiveness for recruitment and retention. Understanding how CEnRT enhances tobacco regulatory research through community and participant engagement in rural settings is important to inform comprehensive FDA regulations. Our AppalTRUST CEnRT approach can also be applied to other public health cohort studies in rural communities or underserved populations. The purpose of this paper is to describe the overarching sampling design and the CEnRT recruitment and retention methods for building the AppalTRUST Cohort.

### 1.4 Aims of AppalTRUST

The overarching goal of AppalTRUST is to build an Appalachian Cohort to investigate the potential or actual impacts of FDA regulatory changes on tobacco product use in rural communities and examine variation based on relative rurality (22). Specifically, AppalTRUST aims to close the gap in rural tobacco disparities by documenting tobacco use behaviors and related factors over time among adults in Appalachian KY to inform current and future FDA regulations. We recognize that mistrust in rural communities is a common barrier to research (16). Our CEnRT approach is designed to gain and maintain trust in rural and Appalachian communities.

To balance rigor and feasibility, we use an innovative dual sampling design, comprising a quota-based non-probability sample (NPS) of 1,000 adults, and an address-based probability sample (PS) of 1,000 adults. Both samples are recruited from two rural catchments in eastern KY, including 5 counties in rural KY and 3 counties in peri-urban KY.

**Figure 1:**
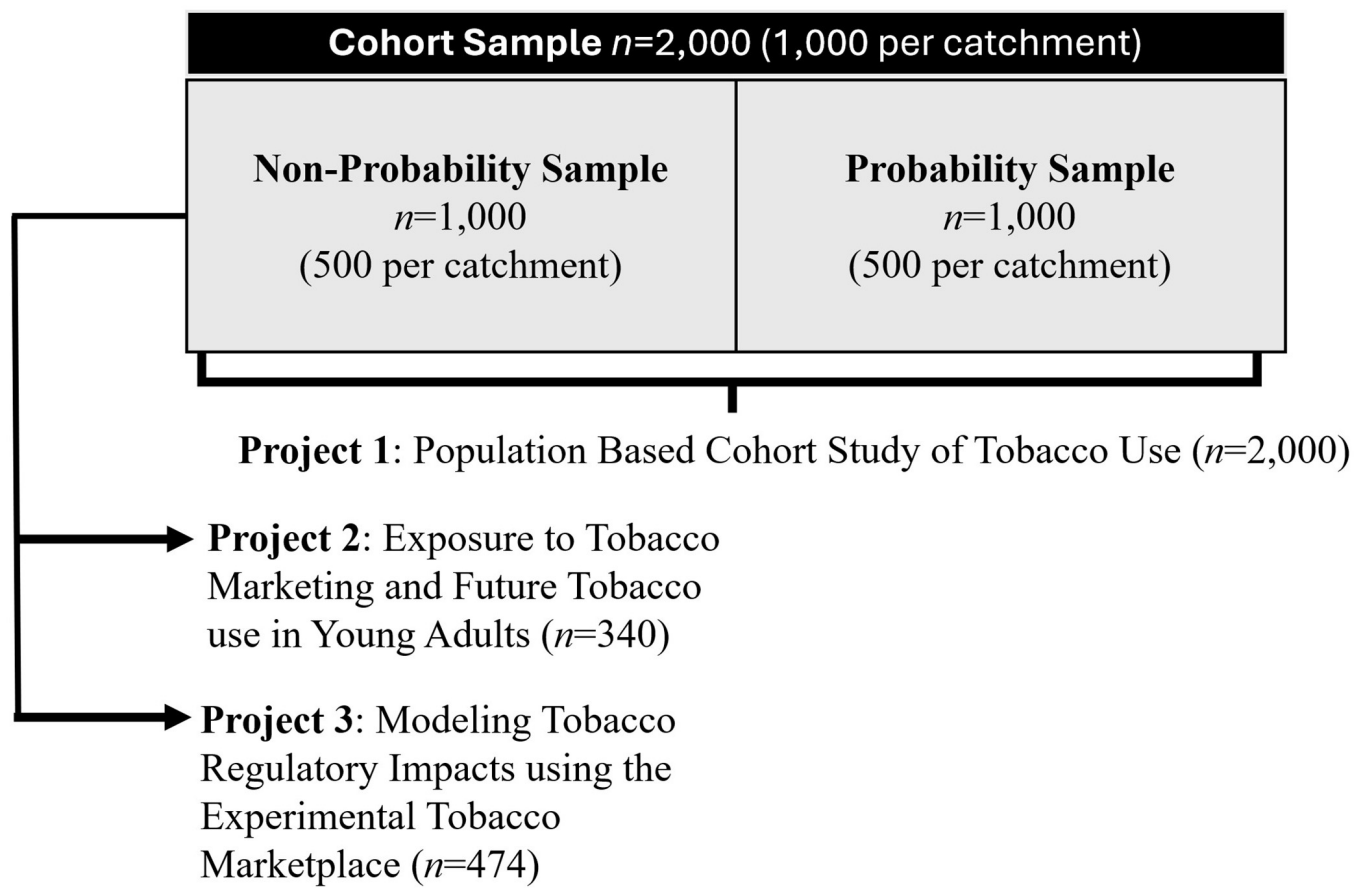
AppalTRUST projects using a cohort design.

AppalTRUST comprises three projects (see Figure 1), and the NPS quotas are set to recruit for these projects. Project 1 uses the full cohort sample of 2,000 Appalachian KY adults (18+ years) to capture estimates of tobacco/nicotine product use behaviors (e.g., initiation, switching, transitions) over time, as well as dependence on, susceptibility to, and beliefs about tobacco/nicotine. Project 2 comprises 340 18–24-year-olds to examine the extent to which marketing exposure, tobacco use behaviors, and attitudes toward tobacco products differ across rural communities and the extent to which FDA regulatory actions can potentially reduce use of tobacco among young adults in these areas. Project 3 comprises 474 participants, 21 and older, who currently use tobacco/nicotine to assess the impact of proposed tobacco product regulations using behavioral economic methods to characterize the consumption of tobacco products within the complex nicotine marketplace of Appalachian KY. This paper focuses on the overarching Cohort recruitment and retention methods that support all three AppalTRUST projects.

## 2 Methods

First, we provide an overview of the AppalTRUST methods and then describe the CEnRT model and strategies in detail. AppalTRUST has unique cores, or shared services, that support the three projects and the center. The community outreach and participant engagement (COPE) Core leads recruitment and retention of the AppalTRUST Cohort. COPE comprises a team of experts in Appalachian culture, community-engaged research, and innovative and rigorous sampling and recruitment strategies, as well as a connected, highly trained local field team who have lived in the region most of their lives for on-the-ground activities. The local field team is key to building trust as they are acutely aware of the cultural and logistical complexities of recruiting a sample that reflects the variation of rurality in Appalachia (See Section 2.1, Building trust and cultural relevance). COPE works closely with the Biostatistics and Informatics Core (BIC), comprised of experts and staff with extensive experience developing online surveys and databases, tracking participant engagement and response rates, managing and analyzing data, and ensuring ongoing data security. Together, COPE and BIC provide centralized management of the AppalTRUST Cohort to support all three projects using our inter-connected CEnRT recruitment and retention model. This approach leverages the long-standing and trusted network of local partnerships and locally hired staff who have built and maintained relationships over time in the region.

**Figure 2:**
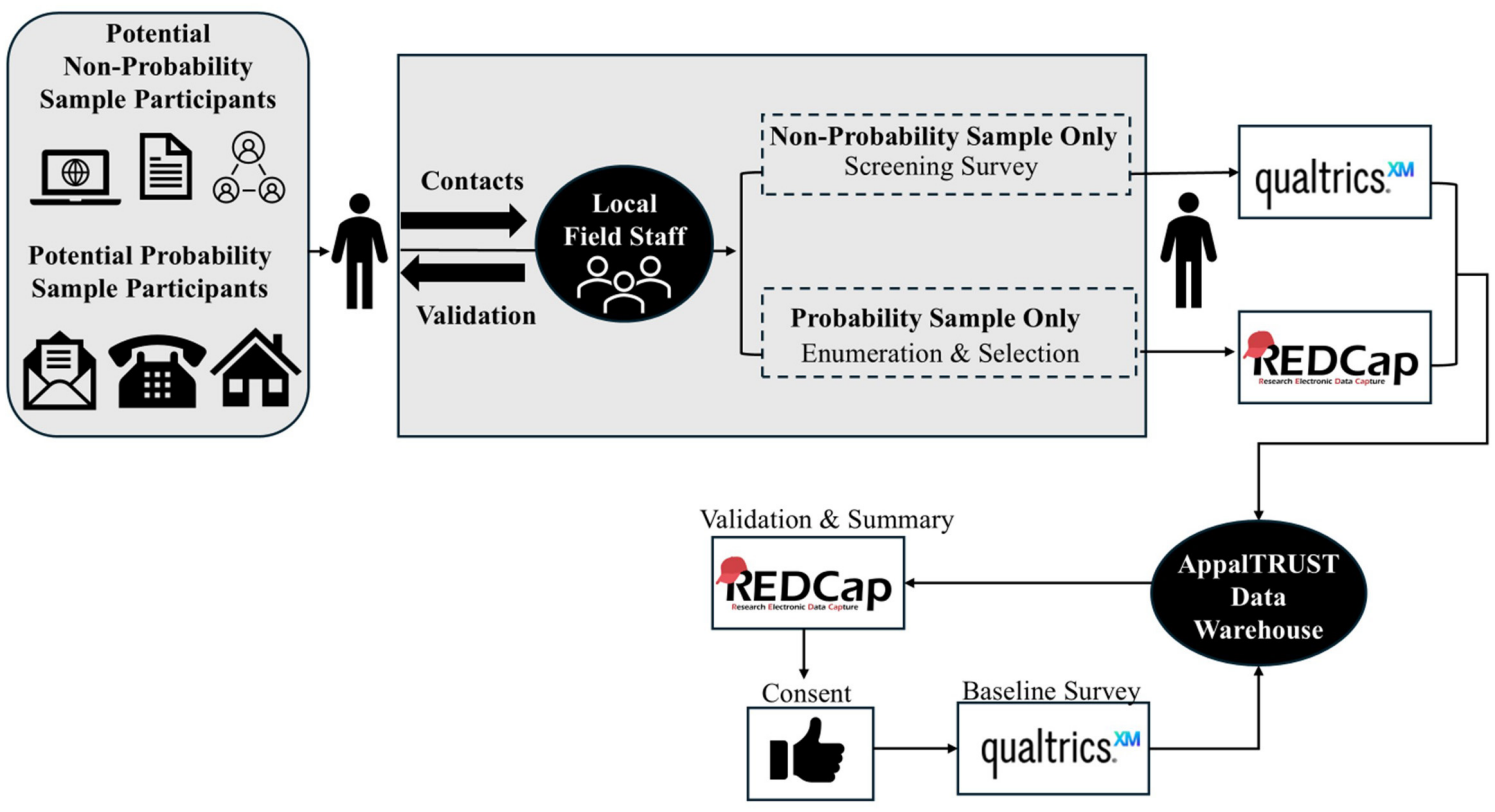
Overview of AppalTRUST components & CEnRT approach.

Our local field team integrates participant engagement strategies (e.g., authentic and consistent communication that meets people where they are) and technology (e.g., online outreach, various forms of digital communication, and online reporting systems) to foster trust and relevance with an underserved population (23) (see Figure 2). We use online Qualtrics forms for all quantitative participant-facing data collection, including screening for eligibility, consenting to participate in the study, and biannual surveys. We use research electronic data capture (REDCap) for all staff-facing data collection, participant e-consent, and participant tracking. We use Apache Airflow to allow communication between the REDCap and Qualtrics systems to create unique survey links for each participant and generate email invitation lists for survey participation (Shamblen, S. et al., manuscript under review). All study materials and procedures were approved by the institution's human subjects review board. Participant data are housed within REDCap, a secure platform for storing data and generating reports (21).

### 2.1 Community-engaged research + technology model

Our CEnR approach relies on local field teams to build trust and cultural relevance as they recruit, enroll, and retain study participants in Appalachia. We describe our adapted model by integrating technology (T) for efficiencies and trust building, expanding our acronym to CEnRT. We integrate technological components (e.g., immediate screening for eligibility and sending consent and survey links through various digital formats) with participant engagement practices (e.g., consistent contacts, reminders about next steps in the process). Other CEnR studies have reported the value of integrating information and communication technology for best practices (21, 24). Here, we highlight the technological components that facilitate authentic participant engagement and enhance recruitment to and retention in an Appalachian cohort.

Our overarching CEnRT model for guiding recruitment and retention relies on merging participant engagement and technological methods using four inter-connected elements: (1) build trust and cultural relevance; (2) address barriers to participation in research; (3) focus on ethics and confidentiality; and (4) foster ongoing and consistent collaboration between the scientific and field teams (Table 1).

**Table 1:** AppalTRUST CEnRT model, strategies and technology methods.

Model elements	CEnRT participant engagement strategies	CEnRT technology methods
Build trust and cultural relevance	•Hire, train, and engage a local field team with culturally relevant knowledge and history of region. •Integrate feedback from local stakeholders, influencers, and organizations.	•Integrate information technology (e.g., online outreach), analytics, and visualization tools to sustain engagement through all stages of recruitment and retention. •Incorporate technology to allow for preferred management/storage and culturally relevant methods of contact and retention. •Use a trusted website URL, university branding, regionally appealing logo, and participant-facing communications for screener and surveys to foster culturally tailored messaging, legitimacy, and authenticity.
Address barriers to participation in research	•Engage participants as community experts throughout the study. •Meet community members where they are. •Allay concerns and address the sources of mistrust that community members may feel. •Inform participants that Appalachian representation in tobacco regulatory science research is an opportunity for them to be represented and have local voices heard. •Use multi-method recruitment methods that are relevant to the population of interest (e.g., community events/festivals, direct engagement, targeted media campaigns, and promotional materials). •Go to where the target population is (e.g., community colleges, convenience stores).	•Use of multiple statuses in REDCap maximizes efficiency and effective contacts by indicating where the participant is in the process and their preferred method of contact - the right method, at the right time, and the right message (the “All Method”). •Use of analytic indicators for participants considered “high priority” (e.g., survey due in 14 days), or “high needs” (e.g., low literacy, home technology or internet issues, lack of existing email) fosters timely contacts and retention in the research. •Information technology and visualization tools used for route planning and scheduling (combined with participant study status) to lower barriers to participation. •Field staff provide technological study devices (e.g., tablet) to complete surveys as needed. •Field staff meet participants in the community if residence is not an option (e.g., lack of internet, unhoused). •Use assorted technology to interact with participants (e.g., ClerkChat, text, voice calls, email) to ensure higher rate of contact and allow participants to use preferred method. •Use a technology-assisted screening process that includes fraud detection of ineligible participants (i.e., youth, those outside of catchment) and a non-judgmental approach (e.g., explaining the ‘why' of eligibility and ineligibility).
Focus on ethics and confidentiality	•Help participants understand the importance of being a research participant and the human subject's protection elements of research. •Review consent forms via video or in person to foster trust and clarify study protocol. •Staff use consistent contacts, reminders about next steps in the confidential process.	•Use of different modes for consent based on participant preference (Facetime, Zoom, Teams, Google Meet) cultivates trust and inclusion of underrepresented populations. •Staff immediately screen for eligibility and send consent and survey links. •Participants complete surveys/consents/forms on preferred devices (tablets, phones, computers). •Consent process informs participants that their data are secure and will remain confidential. •Staff inform participants in a straightforward way that we collect data and handle data using industry standard software, built with data security and provenance in mind (i.e., REDCap, Apache Airflow, and Qualtrics).
Foster ongoing and consistent collaboration between scientific and field teams	•Recognize field team as local experts. •Review all draft plans and finalize methods together in real time. •Communicate daily to jointly navigate challenges and celebrate successes.	•Monitor, track and pivot when needed, with review of caseloads, high need participants, enrollment, and retention rates. •Track enrollment and retention rates as a whole and by staff member and catchment area, and adjust as needed (assignments, frequency of contacts, person making contacts, recruitment methods, locations) •Use data dashboards to provide real-time updates to the field team, the research team, and project stakeholders about data collection processes, and potential areas for improvement and course correction.

#### 2.1.1 Build trust and cultural relevance

As a critical first step, we recruit, hire, train, and authentically engage a local field team composed of long-standing community members (17) To identify effective staffing approaches, we consult directors of similar regional initiatives and distribute a tailored recruitment flier through local organizations, schools, coalitions, colleges, and universities, as well as directly to trusted community members recommended for their relevant expertise. Field team members maintain CITI Human Subjects Research certification and complete training in research security, conflict of interest, cybersecurity, and harassment prevention. We also conduct in-person trainings lasting 8–16 h that focus on protocols, methods, and implementation strategies, taught and then refined collaboratively based on team feedback regarding regional best practices, field experience, and enrollment progress.

These trusted research staff naturally use local dialects, cultural references, and traditions when engaging with participants. They acknowledge the distinct history and challenges faced by rural Appalachian populations, including those associated with living in tobacco-growing regions, to show respect for the region. Importantly, they understand the potential challenges they will face as the ones consenting and collecting data, and they can suggest workable solutions for every challenge. This knowledge is also critical to informing culturally tailored messaging and methods for successful participant engagement, recruitment, consenting, and retention. When the field staff say they are ‘from here' and ‘live in County X,' participants are put at ease and are more likely to talk with them. Hiring the right local staff ensures a team that can effectively engage and integrate feedback from local stakeholders, influencers, community-based organizations, and Community Advisory Boards (CAB), or research coalition boards (21).

#### 2.1.2 Address barriers to participation in research

We engage community members early on and throughout the study to learn their concerns and challenges to participate in research. We recognize that each community is different, and listening to community voices and incorporating their suggestions enhances research protocols and fosters success in recruitment and retention. In this process, we are deliberate in communicating each person's value as a community expert. We conducted stakeholder interviews and CAB meetings early in the establishment of AppalTRUST to listen to community perspectives on our suggested approach and methods, and we maintain communication through regular meetings and rapid response feedback requests. We learned about the many nuances in Appalachia, including how different age groups and regions receive their information and the sources of mistrust toward researchers. We learned that a multi-method approach to communication was best. As such, we use mailers, print materials, and swag (promotional products) with QR codes specific to the catchment area (e.g., peri-urban, rural) to provide contact information and/or direct participants to a link to complete the screener. We then follow up with consistent messaging via texts, phone calls, and e-mails (up to 4 attempts with each method before communication is paused and then resumed later). Our field team refers to this multi-channel, participant-led contact protocol as the “All Method”, a deliberate strategy in which mailers, texts, phone calls, e-mails, and in-person contact are deployed in flexible combinations to reach participants through their preferred communication channel. The “All Method” is a deliberate approach to meet people where they are and allows participants the opportunity to engage in a way best suited to them. We also respect community members who wish not to be contacted further.

#### 2.1.3 Focus on ethics and confidentiality

All participant-facing materials and scripts, as well as all research protocols, are submitted to the IRB of record, FWA00005295. BIC facilitates a process by which all surveys and other data forms completed by the same participant are identifiable through a unique participant number. In addition, a BIC team member plays the role of honest broker, allowing synchronization of forms and surveys from the same individual without compromising confidentiality or having inadvertent mismatches. The honest broker is a neutral team member that serves as a secure, trusted intermediary between protected health information and researchers (e.g., by de-identifying data or samples) to protect patient confidentiality. In addition, we design our recruitment messages and materials with ethics and confidentiality at the forefront. Rural Appalachian populations often feel as if their experiences and values do not matter, and they may feel that they are being used as research subjects for observations or interventions that will not benefit or serve them (21). We are deliberate in helping participants understand the importance of participating and having their data included to inform FDA regulations on tobacco products, marketing, and sales. In this spirit, we use this direct message on promotional materials: “Make your voice heard!” The field staff also regularly communicate the critical importance of local voices, and the value we place on participant confidentiality and safety. Field staff review consent forms individually, either on video chat or in person, to ensure participants understand the purpose of the study, how we will use their data, and how we keep their identity and survey responses safe.

#### 2.1.4 Foster ongoing and consistent collaboration between scientific and field teams

We prioritize ongoing collaboration between the field staff and the scientific team, as this is paramount to success (17). It is essential that the field team be recognized as experts on the research team and that their concerns, solutions, and ideas are fully integrated into all recruitment and retention methods. To this end, our training sessions are collaborative and interactive co-learning spaces. As a team, we review all draft plans and finalize protocols, methods, and recruitment targets in real time. We hold regular meetings, on-site team retreats, and events to foster collaboration. The scientific leads communicate with the field team daily via Microsoft Teams, navigating challenges and celebrating successes together. We use dashboards to show real-time progress with recruitment, enrollment, consenting, and retention, as well as where there are gaps or lags that need additional support or re-strategizing.

Across these four inter-connected CEnR participant engagement elements, we integrate technological components to ensure sustained engagement with participants through the recruitment, screening, consenting, and enrollment processes. Due to our dual sampling design, we developed separate processes for recruitment and retention for the NPS and PS. These processes are described in detail by component in Sections 2.2.1–2.2.6.

### 2.2 Building the Appalachian cohort: an innovative sampling design

**Figure 3:**
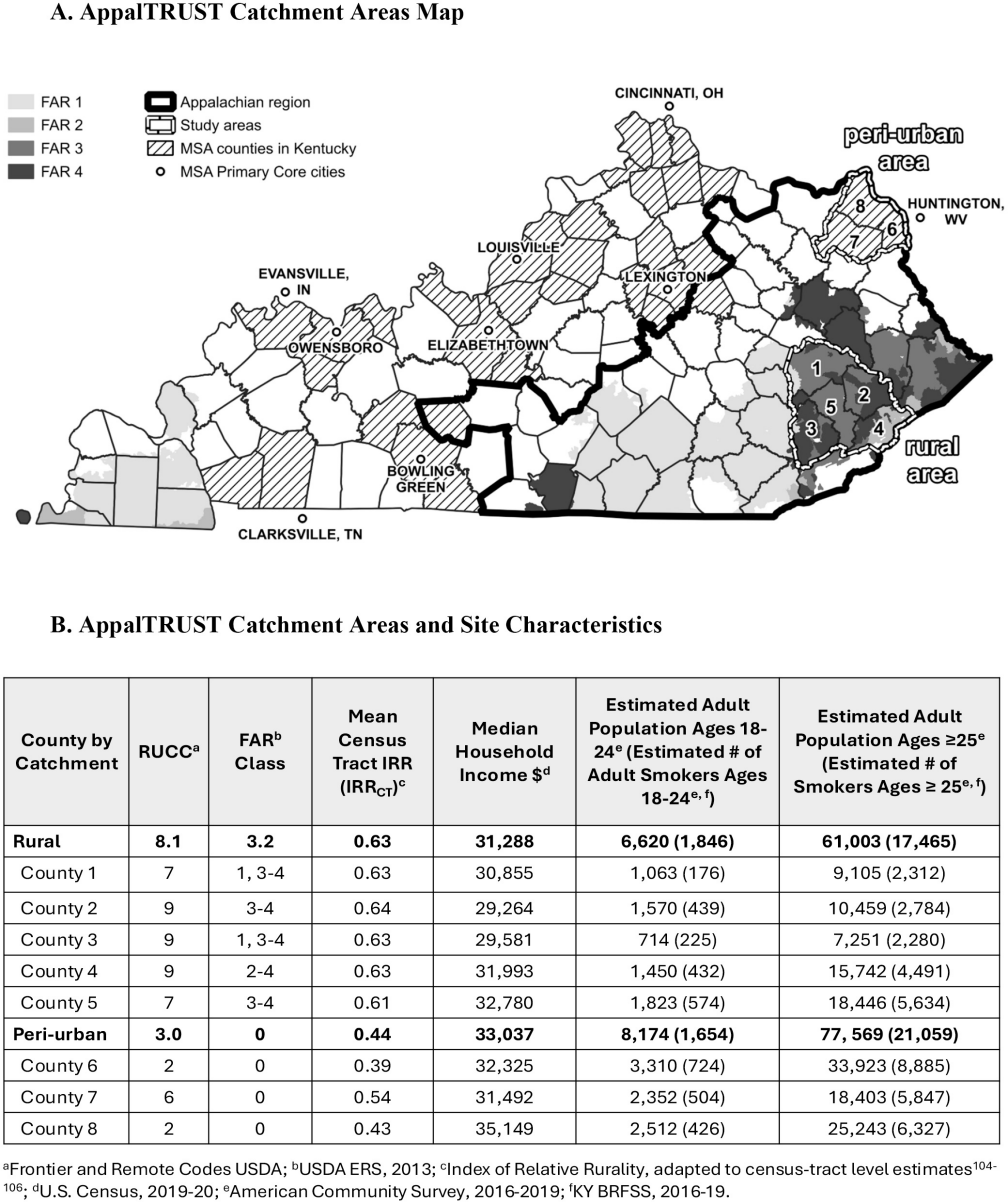
AppalTRUST map, catchment areas, and site characteristics.

We launched the AppalTRUST Cohort in July 2024 from eight rural counties in two catchment areas. We designed the sampling frame to allow assessment of the association between rurality and study outcomes (see Figure 3). Rural areas can be defined by several factors including population size and density, remoteness, and extent of urbanized area. We extend this definition to cover different levels of remoteness as well as peri-urban (urban adjacent) areas. To this end, we include five contiguous counties in rural Appalachian KY and three contiguous counties in peri-urban (22). We use the Rural Urban Community codes and Frontier and Remote Areas codes (25) to categorize the rural/urban continuum. We also use the Index of Relative Rurality (IRR) to examine the range of rurality at the zip code and census tract levels (26–28).

Based on the 2020 5-year American Community Survey estimates, (29) the eight Appalachian KY counties represent a total of 153,366 residents ages 18 years and older, 10% between ages 18–24 (14,794). The sample is balanced on sex and is homogeneous on race and ethnicity. One-fifth is at or below the federal poverty level. Residents are nested within 78,840 households with an average of 2.43 persons per household. The majority (76%) of households have broadband internet access (29). Using combined behavioral risk factor surveillance system (BRFSS) data (2018–2020) for Appalachian KY counties, (3) we estimated that 27% of the study sample would be tobacco cigarette users, 10% would be e-cigarette or vape users, and 10% would be smokeless tobacco users (30). The two catchment areas represent a range of rurality, allowing us to compare use indicators between rural and peri-urban areas. We use sample weighting to adjust parameter estimates so they are demographically representative of the populations living in the counties.

While recruitment is ongoing, the AppalTRUST Cohort will include 2,000 adults (ages 18+) living in Appalachian KY. As mentioned, our sample comprises two components: (1) a quota-based NPS of 1,000 participants; and (2) an address-based PS of 1,000 participants. Our innovative dual sampling design makes cost-efficient use of the PS — a traditionally challenging and resource-intensive method in rural and Appalachian regions — to calibrate estimates from the NPS. Specifically, we will use a quasi-randomization approach with propensity methods to model selection into the NPS through exogenous variables (e.g., mental and physical health, disability status) that are measured in both samples and are correlated with tobacco use outcomes. The NPS and PS data will then be combined and calibrated, via a raking adjustment, using population control totals from the American Community Survey for sociodemographic variables, such as sex, age, education and income, yielding a combined analytic dataset appropriate for population-level inference, consistent with established methods for integrating NPS and PS for survey inference (31–34). This approach has been shown to effectively reduce error variance in estimates derived from non-probability samples and has been successfully applied in health research contexts analogous to the present study, including population-based surveys of cancer catchment areas (32). The combined NPS+PS dataset will be particularly valuable for analyses of small subgroups (e.g., young adults, product switchers) for which a probability sample alone would not provide sufficient statistical power (31, 33, 35).

Our study uses a longitudinal cohort design with rolling recruitment to follow the Appalachian Cohort for 4 years, collecting data every 6 months. Depending on when they enroll, participants are invited to complete 2–7 surveys. Data is collected through an online survey template that standardizes tobacco-specific items across waves while allowing rapid addition of new questions in response to changes in the tobacco product and regulatory landscape. Assuming 80% retention, we expect to retain at least 1,600 engaged participants by study end. The following sections describe recruitment, screening, consent, and enrollment procedures for the NPS and PS samples.

#### 2.2.1 AppalTRUST NPS recruitment and screening methods

For the NPS, we generate community awareness and interest in AppalTRUST largely through community outreach, community event attendance, and culturally appropriate promotional materials (e.g., flyers, palm cards, coasters, swag). Potential NPS participants are recruited through various means such as community festivals, events, health fairs, pop-up table promotion, direct recruitment, and strategic promotional placement of flyers, palm cards, and coasters. We also recruit from local workplaces like restaurants, recreational facilities, or ‘pop up events' at farmers' markets or gas stations. As young adults are hard to engage in rural Appalachian areas, our trusted local field team and CAB members suggested we partner with the community and technology schools to help us recruit for P2, which has been highly successful. Field staff also visit coffee shops with printed materials and appealing swag (e.g., water bottles, fidgets) to make personal contact with potential young adult participants. Finally, our CAB members and field team suggested we tailor the direct message on promotional materials for young adults: “My Future, My Voice.”

Interested participants scan a QR code or enter a URL in their web browser to participate in an initial screener survey that determines eligibility for all three AppalTRUST projects (see measures in Section 2.3). Upon completion of the NPS screening survey, an automated workflow determines inclusion. Specifically, participants are screened out of the NPS if they: (a) indicate a different age on two items inquiring about age (one open-ended and one ordinal forced-choice item); (b) enter an invalid address, as determined by using the Melissa address verifier (36); (c) have a response IP address that is suspicious (i.e., non-KY IP address); (d) are inattentive (i.e., leaving the survey prematurely); and /or (e) report characteristics that match a currently enrolled participant (i.e., address, name, email, and phone number).

We do not further contact ineligible participants nor those in which eligibility is unclear (e.g., age not indicated; missing address). When automatic screening results are unclear, the field team manager examines these cases to determine if the participant should be contacted for further recruitment efforts and cautions field staff about any suspicious or incomplete data. Duplicate screeners are the most common instances of possible fraud. Participants are flagged for duplicate contact information, including names, email addresses, phone numbers, and mailing addresses. Some cases are easy to resolve, such as when names are similar but not identical and all other demographic identifiers are unique. Others require further review. In those cases, field staff follow up with the participant, and video consent is required (see 2.1.2 NPS consent and enrollment). For example, if a participant has the same name and age as another enrollee but different contact information (e.g., physical address or e-mail address), the original field staff member assigned to that enrollee conducts the follow-up to rule out duplicate participants.

#### 2.2.2 NPS consent and enrollment

Participants who successfully screen for the NPS study are assigned to a field staff member who implements the “All Method” (described in Section 2.1) to contact them for consent and enrollment. In cases where a participant cannot be reached, we pause screening efforts and then implement the All Method again 30 days from the last contact. If a participant still cannot be reached after another 4 attempts, they are assigned a final disposition of “unable to contact”.

We offer different video call modes (e.g., teams, zoom, facetime, and google meet) for electronic consenting (e-consenting) to ensure the participants are comfortable with the format. Teleconferencing with participants during e-consenting helps create a human connection and fosters a relationship that supports study retention. During e-consent, we inform participants of their eligibility and provide them with a study overview, confidentiality measures, and communication about the survey and payment processes. We ask participants to provide their birth date, which allows their payment debit card to be activated. Lastly, participants e-sign the consent form, and we e-mail a copy to them. We provide them with study contacts should they have questions or concerns. If applicable, we also inform participants about their eligibility for the other AppalTRUST projects, the purpose of these projects, their potential role in the research, and potential incentives. This “warm hand off” promotes awareness about future contacts from study staff as well as enhances participant trust and interest in the research. We make exceptions to e-consenting in special circumstances, such as personal connection to the participant, meeting the participant at a recruitment event, or barriers with technology.

Once field staff contact the participant during the consent process, additional methods of contact are obtained (i.e., social media, alternate phone numbers, mailing address, secondary person who would be considered a reliable contact, etc.). Additionally, field staff confirm participants' preferred method of contact. Having secondary contacts as well as a preferred method of contact allows field staff to be more effective and efficient with their communication attempts, and also ensures we can maintain contact for follow-up surveys. To date, the majority of participants (62%) have selected text messaging as their preferred method of contact, followed by e-mail (27%) and phone call (12%).

Once the consent process is complete, we email and text participants a unique URL that takes them to their survey. Each survey takes 30–45 min to complete. Participants are paid $50 for each survey completed. For the initial payment, participants are mailed a Greenphire debit card with $50 loaded. This is also a method to verify their home address. Subsequent payments are simply loaded onto the same card.

#### 2.2.3 NPS participant engagement tracking and retention

Daily, the field staff review REDCap reports that provide disposition codes (see Table 2) for each assigned participant, detailing which participants need to be contacted that day and where they are in the survey completion process. The scientific and field teams co-developed the disposition codes to ensure processes and codes were intuitive and helpful for tracking. For cases with a non-enrolled terminal disposition, we use the American association of public opinion research (AAPOR) (37) disposition codes to foster easy calculation of response rates. Non-AAPOR codes will be converted to AAPOR codes for final dispositions.

**Table 2:** AppalTRUST disposition codes for screening NPS and PS study participants.

AppalTRUST Disposition Codes
AppalTRUST disposition codes for participantengagement
01. Active
02. Consented
03. Paused
04. Declined
05. Enrolled (completed initial survey) − No action needed until follow-up surveys
06–12. Completed 6-month follow-ups^*^
13. Quota met
14–20. Scheduled to go out^*^
21–27. Due in 30 days^*^
28–34. Due 7 days^*^
35–42. Overdue^*^
28. PS pre-screener (selection)
29. PS mailer sent
30. PS withdrawn
AppalTRUST AAPOR disposition codes for non-enrolled (based on AAPOR, 2023)
2.1 Refusal
2.2 Unable to contact
2.3 Unable to communicate with participant
3.1 Technical or delivery barriers, so never determined eligibility
3.2 Screener only partially completed, so eligibility not known
4.3 No such address/person (PS Only)
4.5 Not a housing unit (PS only)
4.1 Screened out
4.7 Did not meet initial eligibility criteria
4.8 Quota filled or duplicate sample
3.9 Other: eligible initially, then determined ineligible
4.9 Potentially fraudulent
4.9 Other: ineligible

^*^When multiple numbers are listed (e.g., 6–12), multiple waves (up to 7) are represented by the code.

As part of tracking participant engagement, field staff record the result of each contact attempt (i.e., disposition code) and if/when they should follow up with the participant. To boost participant engagement tracking, field staff sort REDCap reports by disposition code to indicate where the participant is in the process, such as “ready to be contacted”, “consented, not enrolled,” “paused,” “due in 14 days,” etc. (See Table 2). Codes inform field staff about the survey wave (W1, W2, W3, etc.), as well as how long the participant has before their 30-day window expires. These codes allow field staff to establish case prioritization and foster a standard daily routine. Field staff sort their reports by date to easily see the contact attempts needed each day. Every other week, the primary COPE scientist and the field team manager review a summarized Excel file with disposition and status code frequencies by catchment area and staff member to help set priorities and establish needed supports. This tracking mechanism is critical for timely and effective communication with participants based on their preferred method of contact.

An important principle we follow is that retention begins during the first conversation with participants. Each field staff is deliberate in showing passion for AppalTRUST, as well as compassion for each participant with whom we engage, valuing their time, questions, and their concerns. We are also committed to maintaining participant engagement between surveys with hand-written birthday cards, hand-written postcards to request updated contact information, emails and texts with holiday wishes, reminders to update contact information, and reminders to complete surveys each week until they are due. While everyone on the team supports these efforts, we adjust protocols for participants who have not completed their follow-up surveys with less than 2 weeks remaining until their due date. Specifically, the field staff member originally assigned follows up to remind them of their initial contact, the purpose of the study and their importance to the cohort, and to encourage them to complete the survey. To boost retention, we use a unique status code (“due in 14 days”) to alert the field staff to prioritize these contacts. We use other best practices to boost retention (38) to maintain the cohort including: $50 for each survey completed, and not excluding participants from the cohort due to missing one survey or withdrawing from the other AppalTRUST projects.

#### 2.2.4 AppalTRUST PS recruitment and screening methods

We hired Marketing Systems Group, a company specializing in sampling and technology for survey research, to obtain a random address-based sample of about 2,000 households from the catchment areas to ensure we reach our target sample of 1,000. We provided the vendor with the exact number of random selections needed from each stratum (i.e., 50% in each of rural and peri-urban and proportional allocation for counties within rural/peri-urban strata). Our stratified random sample of households for the PS excludes vacant addresses and PO boxes. Within each stratum, we randomly select addresses from the United Stated Postal Services Delivery Sequence File. We pull these samples in smaller, replicate samples (i.e., 300 households at a time, or 150 per catchment) for a few reasons: to (1) ensure manageable caseloads for field staff; (2) attempt household visits in alignment with the timing of recruitment mailers; and (3) adapt protocols based on lessons learned and track the impact of revised or added strategies.

Our CEnRT recruitment approach for the PS includes tailored, culturally appropriate direct mailers to households randomly selected based on address. We hired United Direct Solutions, a local direct mail and print company, to deliver our mailers due to their understanding of the area and the challenges with mail delivery in hard-to-access areas. Selected households receive four mailers, in which the first and third mailers are postcards explaining the purpose of the study. The second and fourth mailers are letters with a similar message but more study details, and a $2 unconditional incentive is included in the second mailer. All mailers ask recipients to contact our team, either by getting in touch with the field staff directly by text, e-mail, or phone, or by using a QR code to complete a contact information form. All mailers and scripts used in recruitment are co-created with our local field team and CABs, who understand the regional landscape, identity, and concerns about research, as well as the historical relationship Appalachian KY has with tobacco.

After the first two mailers have been sent, field staff attempt to contact households by making up to four home visit attempts, and another 4 attempts by phone (calling and/or texting) to those with known phone numbers. After these attempts are made, households are assigned a final disposition code of “Unable to Contact”. About 16% of our sample respond directly to the mailers, and only one-third have working phone numbers from the vendor, increasing the need for home visits. Since human connection is critical for minimizing the homeowners' reluctance to open the door and / or participate in our study, field staff follow several key strategies to foster trust-building, familiarity, connection, and engagement. These strategies include: (a) making home visits at varying times of day, including evening and weekend visits to ensure they reach household members at a time they are available and feel safe; (b) reducing time between visits (e.g., 4 visits within 2 weeks) to foster familiarity; (c) letting household members know that they are ‘from here' and ‘live in County X' to build trust; (d) going out in male/female teams to lessen intimidation and foster engagement; and (e) leaving AppalTRUST-branded door hangers and personal notes behind so residents can look further into the study or call/ text to learn more or say they are not interested.

We use integrated AI, GPS, and GIS technologies to identify the most efficient travel routes to maximize time in the field and coordinate the travel routes by county. We develop routes regionally to ensure efficiency, and we also stop by households with participants who are active in the enrollment process to prioritize in-person contacts when possible.

When we reach a member of a PS household, we first verify age (18+) and residence (i.e., living in the household) and then invite them to complete a pre-screening process to select a household member for study participation. Only one household member is eligible for the PS, though others could participate in the NPS. We define “PS household member” as “someone who sleeps in the household at least four nights per week.” All PS household members are listed by their initials and enumerated, and field staff select a member of the household using random drawings from a uniform distribution in REDCap. Field staff then collect contact information for the selected member of the household and begin the consenting process.

#### 2.2.5 PS consent and enrollment

When possible, the consent process is completed in person with the PS. When needed due to time limitations or internet connectivity issues, we use e-consent when back in the office. Field staff follow the e-consenting and enrollment process used with the NPS and the incentive structure is identical (see Section 2.1.2). One variation to the PS consent processes is that we also offer the option of paper consent protocols when participants are uncomfortable with digital communications. In these cases, we leave a signed copy of the consent with the participant and upload a signed copy to REDCap. We store the signed paper copy in a locked file folder until they are mailed to the PI for storage.

Enrollment procedures also vary for the PS if an interested participant does not have access to the internet and/or an internet-enabled device to complete the online surveys. Specifically, if they are unable to access and/or navigate the online surveys, field staff assist with survey completion, either at their home (if they have internet) or at another private location (e.g., local library).

#### 2.2.6 PS participant engagement tracking and retention

We added codes for the PS, including whether the address comprises a vacant or abandoned household (disposition code 4.3) or has no house at all (4.5), as well as whether the household has been sent mailers (11), is in pre-screening phase (12) or is in screening phase (13). These additional codes are unique to the PS component and help field staff tailor their approach and timing for engagement. Otherwise, the field staff use the same disposition codes as for the NPS (see Table 2).

We follow the same retention methods described with the NPS, but the PS sampling design allows us to enhance retention efforts. Specifically, we make additional household visits with: (a) those who request use of our study devices (i.e., phone, tablet, or laptop) to complete the survey at enrollment; or (b) those whom we have difficulty reaching through phone calls, texts, or e-mails.

### 2.3 AppalTRUST NPS & PS key survey domains & brief descriptions

Each project has its own unique survey measures. However, there are overarching survey domains for all projects, including: (a) sociodemographic factors; (b) tobacco/nicotine product use history; (c) current use of tobacco/nicotine products, brands, and flavors; (d) product-specific tobacco/nicotine dependence among those who have used the product in the last 6 months; (e) product-specific susceptibility to use among those who have never used the given product; and (f) cessation history (e.g., nicotine replacement therapy and other support for quitting). We define tobacco/nicotine product use as anyone using conventional (cigarettes, smokeless/snus tobacco, hookah, cigars/cigarillos, and pipes) or novel (e-cigarettes, nicotine pouches, heated tobacco products, lozenges, gum, and very low nicotine [VLN] cigarettes) products including nicotine and/or tobacco.

The sociodemographic items are from the PhenX toolkit (39) and the Population Assessment of Tobacco and Health (PATH) Study (40). All remaining variables are based on or adapted from either the PATH study or NYTS (National youth tobacco study) (41). Table 3 displays the source of the assessment tools used for each domain, and the description gives more detail on the measures including the original source (if applicable). The PhenX Toolkit includes measures for which there is consensus regarding their use in biomedical and public health research, and the tobacco use measures in this curated repository were developed in conjunction with the National Cancer Institute and the Food and Drug Administration (39). Among adult participants in Wave 1 of the PATH study, urinary nicotine metabolite concentrations were meaningfully differentiated across self-reported tobacco product use categories, including combustible, e-cigarette, and smokeless tobacco products, consistent with the validity of self-reported use (42). An evaluation of Wave 4 PATH data assessed the reliability and validity of a subset of items from the PATH questionnaire, including tobacco use measures, with self-reported tobacco use validated via salivary cotinine (43). While reliability and validity of the NYTS items have not been specifically described in the literature, there is considerable overlap of content between the NYTS tobacco use items and the corresponding ones included in PATH. More broadly, research on national survey data has demonstrated the high degree of agreement between self-reported tobacco use items and biomarker data, including for cigarettes and smokeless tobacco, as measured in the National Health and Nutrition Examination Survey (44, 45). To date, investigations of reliability and validity have not been centered on rural or Appalachian participants.

**Table 3:** Key AppalTRUST survey domains and descriptions for the cohort (project 1).

Domain	Assessment tools	Description (including original source of items, if applicable)
Baseline survey		
Sociodemographic characteristics	PhenX toolkit and Population Assessment of Tobacco and Health (PATH)	Gender, sex assigned at birth, date of birth (for age calculation), race, ethnicity, LGBTQ+ status, marital status, income sufficiency, education, working status, health insurance status, military service, and county of residence
Tobacco/nicotine product use history	PATH and national youth tobacco survey (NYTS)	Ever use of each of 13 tobacco/nicotine products (cigarettes, very low nicotine cigarettes, e-cigarettes, hookah, smokeless tobacco, snus, large cigars, cigarillos, little cigars, pipes with tobacco, nicotine pouches, oral nicotine products not for cessation, including lozenges, discs and gum, and heated tobacco): among ever users, how many times used, ever used regularly, age of initiation, age at regular use, was first product flavored, ever completely quit using product
Baseline and subsequent surveys		
Current use of tobacco/nicotine products including flavored products	PATH and NYTS	For each of the 13 tobacco/nicotine products ever used: past 30-day and past 6-month use; these indicators determine 30-day use of broader product categories (e.g., combustibles, ENDS, oral nicotine products). Among current (past 30-day) users, how many days used in last 30 days, regular brand, and use of flavored products (including flavor types of product brand).
Nicotine dependence (among those who have used product in the last 6 months) and susceptibility to use (among never users)	PATH	Tobacco/nicotine dependence measure based on items from the Wisconsin Inventory of Smoking Dependence or Motives, (60) the Nicotine Dependence Syndrome Scale, (61) and the Diagnostic and Statistical Manual (62); 16 items in the total score determined based on Wave 1 PATH data (63) and we created product-specific scales from this instrument; for susceptibility, committed never users respond ‘definitely not' to three items assessing likelihood of use of the given product (1) in the future; (2) in the next year; (3) or if one of their best friends were to offer it to them (64).
Cessation history, including use of nicotine replacement (NRT) and other quitting support	PATH and NYTS	For each product ever used: ever quit, thinking about or intend to quit within 2 months; checklist for NRT/quit assistance use including patch, gum, inhaler, nasal spray, lozenge, counseling, quit line, support group anytime during last 12 months, and use of these during most recent quit attempt

### 2.4 Data analysis strategies and power considerations

We will use descriptive and bivariate analysis methods suitable for weighted survey data, including SURVEYFREQ and SURVEYMEANS in SAS, v. 9.4. (46) To evaluate associations between demographic characteristics, potential risk or protective factors, and use indicators or other outcomes, we will fit multivariable models for both cross-sectional and longitudinal data. Cross-sectional analyses will use generalized linear models with robust variance estimation, whereas longitudinal analyses will use generalized linear mixed models (GLMMs) or generalized estimating equations (GEEs). Survey weights will be incorporated throughout. For binary outcomes, we will estimate prevalence ratios (PRs) and 95% confidence intervals using modified Poisson regression with robust variance estimation, which provides more accurate PR estimates than logistic regression when outcomes are not rare (47). For continuous outcomes such as tobacco dependence scores, we will use linear regression with sample weights via SURVEYREG. Missing data will be addressed through multiple imputation using the fully conditional specification method, with up to 20 imputed datasets (48) pooled using Rubin's Rules (49, 50). Attrition will be handled using wave- or subset-specific survey weights, calculated for each relevant analytic sample of participants (e.g., Wave 1 completers, participants completing both Waves 1 and 2), to account for differential nonresponse across waves. The attrition adjustment will be based on a response propensity stratification, using propensity models that will account for relevant baseline variables (51). Participants who miss one or more waves will remain active Cohort members and will continue to be invited to participate in subsequent waves unless they withdraw from the study.

Because participants are nested within geographic areas, analyses will account for clustering by including catchment or county as a fixed effect or, in longitudinal models, through the GLMM random effects structure. Beyond binary catchment comparisons, subgroup analyses will use the IRR (22) as a continuous measure at the zip code or census tract level to examine whether tobacco use outcomes vary across the rurality continuum represented in the sample. Analyses will incorporate survey weights reflecting the combined NPS and PS sampling design. Section 2.2 describes the weight construction and calibration approach.

These analysis strategies will allow us to estimate raw (unadjusted) and adjusted use prevalence for each tobacco/nicotine product at baseline and changes in product use prevalence over time; adjusted estimates will be evaluated with relevant demographic and psychosocial variables included as covariates. We will examine prevalence ratios for specific tobacco/nicotine products as well as categories of products, such as combustibles (including cigarettes, all types of cigars, and hookah). We will also assess tobacco/nicotine dependence (among current users) and susceptibility to use (among never users) by product type or category, indicators of cessation and attempted cessation, and other tobacco-related outcomes.

To control overall Type I error due to multiple comparisons, we will use a conservative study-level alpha of.01 for papers reporting numerous findings, including summative analyses of all Cohort participants, regardless of enrollment date. For papers focused on fewer outcomes, including interim analyses of participants enrolled by a given date while recruitment is ongoing, we may use the conventional alpha of.05. When possible, we will also report 95% confidence intervals to contextualize observed *p*-values. Because no standard algorithm is available for power estimation in longitudinal multilevel models with binary outcomes, we will use a two-group comparison for a two-level hierarchical design as a close approximation, representing the special case of one fixed timepoint and a rural/peri-urban comparison of a binary outcome. Assuming an intraclass correlation coefficient (ICC) as large as.01 (i.e., typical maximum for community-based surveys, in which participants are expected to be less correlated with one another than students in a classroom), an alpha level as small as.01, and approximately 1,600 participants nested in 8 counties, the power to detect a difference in two proportions will be at least 81%, when the prevalence of an attribute (e.g., cigarette use) is approximately 35% in one region and 21% in the other. For continuous outcomes, assuming the same ICC, alpha, and sample size, the power to compare two means will be at least 87% when the standardized mean difference is as small as 0.35, an effect size midway between small and medium, per Cohen, (52). These estimates are conservative because they are based on a single timepoint rather than the repeated measures included in the longitudinal models, which are expected to increase power. Based on prior studies with this population and similar retention protocols, we anticipate at least 80% retention in the longitudinal study, consistent with retention rates observed to date.

## 3 Anticipated results

We expect AppalTRUST to generate several key and novel findings.

First, our innovative CEnRT approach to recruitment and retention allows us to disseminate detailed guidance to scientists conducting studies with underserved populations. As can be seen in Table 4, 985 of 1,000 NPS participants and 209 of 1,000 PS participants had been recruited as of February 11, 2026. A total of 3,287 individuals completed the NPS screener; 577 were manually screened out, most often because of suspected bots or repeated screening attempts to obtain the incentive. Approximately one-third of those screened for the NPS (*n* = 1,135) were excluded because project quotas had been met. Participation was declined by 508 NPS participants and 335 PS participants. Among those screened to date, 82 NPS individuals and 556 PS individuals remain in active recruitment by field staff (1,100 addresses are included in PS sample as of February 2026). Among all participants ever enrolled, only 60 NPS participants and 7 PS participants have explicitly discontinued participation in the longitudinal study.

**Table 4:** In-progress recruitment and enrollment status.

Recruitment and enrollment status description	Non-probability sample	Probability sample^*^
Target sample size	1,000	1,000
Screened	3,287	209
Manually screened out (e.g., fraudulent, already participating)	577	–
Enrolled	985	209
Declined participation	508	335
Quota met (based on other project sample needs)	1,135	–
In process	82	556
Enrolled but later withdrew	60	7

^*^1,100 homes had been selected and sent recruitment mailers at this time.

Recruitment and retention numbers reflect recruitment and enrollment status as of February 11, 2026 when we developed this paper.

Second, our cohort and sampling strategy permits us to characterize tobacco use patterns and trends in rural Appalachia, making comparisons along the rural to peri-urban continuum. The CEnRT recruitment and retention strategies help us build and retain an adult cohort from Appalachian Kentucky, while the dual sampling design ensures the resulting estimates of tobacco/nicotine use will be representative of the population in this region. In addition, the multiple, integrated projects will inform our understanding of tobacco/nicotine use patterns, marketing exposure among subgroups of young adults, and potential impact of regulations among individuals who use tobacco/nicotine daily in Appalachia. As one example, we will link Project 2 marketing exposure data with use indicators for tobacco/nicotine products or product categories (from the Project 1 survey) to assess whether these are associated. The longitudinal design will allow us to examine whether marketing exposure predicts future attitudes, perceptions, and behaviors. We expect these findings to inform FDA tobacco/nicotine product regulations with the goal of reducing tobacco disparities in rural communities. By focusing on this understudied population, we will gain valuable insight not only into patterns of use and co-use of tobacco/nicotine products in this region, but also the factors that are associated with use. Our team is currently working on analyses to develop papers on baseline results from all projects. We anticipate publishing baseline results and the NPS STROBE (53) in 2026–2027, and additional methods and outcomes papers related to the PS in 2027–2028. Results of these analyses will help inform FDA about regulations that would be most likely to encourage cessation or prevent initiation of tobacco/nicotine products in this population disproportionately affected by tobacco/nicotine use and its sequalae. Results will also inform TRS and public health researchers on effective strategies for conducting studies in rural Appalachian populations.

## 4 Discussion

Our novel CEnRT methods minimize the intractable challenges to recruiting and retaining cohorts in underrepresented populations like rural Appalachia (21). The CEnRT approach overcomes common barriers of (1) mistrust (16); (2) reaching participants living in remote locations who often lack access to the internet, phone, (8, 15) and digital communication; (3) low reading and technology literacy (15); and (4) the need for regular, seamless, and flexible communications between field teams and participants as well as between field teams and scientific teams (17).

Our four-pronged CEnRT approach uses tailored, interconnected methods to build trust, strengthen cultural relevance, and reduce barriers to study participation. We employ local field staff who speak local dialects and understand community traditions and cultural references, enhancing recruitment, consent, enrollment, and retention. Our “All Method” outreach strategy addresses mistrust by treating participants as community experts and engaging each person through the communication approach that best fits their needs. Because rural participants vary in access to communication technologies, the All Method meets people where they are, combines multiple outreach options, and emphasizes the importance of each participant's voice in the research. Offering multiple communication options during consent also helps address limited reading and technology literacy (24).

Sustained collaboration between the scientific and field teams is essential to building a cohort grounded in trust, tailored outreach, and literacy-sensitive engagement. Scientific leads and field staff jointly monitor progress in real time, enabling the team to respond to challenges and recognize successes. Our CEnRT approach prioritizes authentic, ongoing collaboration with communities while using technology to integrate best practices in community-engaged and survey research. These technology-supported processes also strengthen recruitment and retention by providing consistent support to trusted local field staff. Our CEnRT method goes beyond other CEnRT approaches (54) by tailoring and localizing participant engagement strategies and embedding technology in all aspects of recruitment and retention. Our CEnRT model is relevant and useful for community-engaged researchers in nursing and public health, as well as scientists and practitioners who engage with underrepresented populations. Addressing rural health priorities using our CEnRT model could guide research in areas such as maternal child health, substance use disorders, mental health, and cancer screening to reduce rural disparities. Based on the recent whole health, whole communities movement (55), our CEnRT model is particularly timely and usable for those conducting cross-sector, community-embedded participatory research in rural communities.

High tobacco use prevalence, low cessation rates, and limited access to healthcare in rural America present known disparities. As a result, rural residents suffer disproportionately from high rates of persistent tobacco-related chronic disease and premature death (56). Yet little is known about the impact of tobacco regulations in rural areas. The FDA implements policies to regulate tobacco manufacturing, marketing, and distribution of all tobacco products, and they support research through the TCORS Centers like AppalTRUST. There is an opportunity to reduce rural tobacco disparities by applying novel and effective community-engaged research methods in tobacco regulatory science. Beyond methodological contributions, the AppalTRUST Cohort is uniquely positioned to inform FDA decisions on flavored product restrictions, nicotine-reduction standards, and point-of-sale marketing regulations in rural populations, where exposure patterns and product preferences may differ markedly from urban samples.

### 4.1 Advantages and limitations

There are multiple advantages and challenges to our CEnRT approach. First, recruitment in a rural terrain is a challenge, especially given limited staffing and resources. For PS recruitment in the home, houses are spread out and the roadways may be unpaved or non-existent. The routes may be very long and winding, increasing travel time to the home depending on weather and road conditions. Further, catching people while they are at home can be challenging. While we ask staff to be flexible with their work times (e.g., taking off fridays to work saturday), there are only so many evening hours and weekends we can expect staff to be available for work, and many participants prefer to be contacted during these times. We also experience the seasonal impacts of flooding, ice, and snow, creating project delays. The advantage of having a field team who are long-standing members of the community is the opportunity to connect our research team with disaster relief partners, community service, and personal donation opportunities for disaster relief in rural communities.

Second, fewer recruitment opportunities in rural communities present limitations. Young adults can be hard to find in Appalachia, given this is a transient population with a 6% migration outflow (i.e., people leaving the area) from our 8-county catchment area (57). Remote rural communities also hold fewer community events, limiting the opportunities for NPS recruitment. Further, events may attract people from out of town who screen but are not eligible for the study. To boost NPS recruitment, we distribute ‘palm cards' and make personal connections in community locations (e.g., coffee shops, vape/tobacco shops, tattoo parlors). Regarding PS recruitment, with our address service, we have recent phone numbers for less than half of the sample (43% with cell phone only and 26% with home phone only), and about one-third of those are out of service. This requires us to make the majority of PS contacts via home visits. There is also the limitation of addresses in the sample that are abandoned or vacant (about 14% of obtained sample).

Third, in the spirit of inclusivity, for the NPS, we make an effort to reach out to commonly underserved populations such as those living in temporary or transitional housing, or unhoused persons who attend our community events. Thus, a portion of our participants are unhoused or living in subsidized, income-based housing units. Unstable living conditions, and associated mental and physical health risks, can cause challenges with retention due to transitional addresses and phone numbers, or medical challenges which keep individuals from participating or engaging with the local team. To help allay this, we regularly update contact information, communicate via social media, send frequent reminders, and invite participants back to the Cohort if they have missed a survey.

Fourth, the screening and consent processes provide multiple challenges. Interested participants may attempt to enter the screening survey more than once, creating the need to monitor fraudulent activity. Sometimes participants expect payment after taking the screening survey, thinking the screener is the same as the data collection questionnaire. In response, we revised our language to review the time required for screening (3–5 min) vs. the survey (30–45 min). For NPS consent, we insist on a video call to verify the participant's identity; however, some participants are reluctant to join via video. Participants will typically join a video call for just a moment and then turn it off to view the consent form that field staff are sharing on screen. Field staff remain on camera/video, so their faces are visible. We also have difficulty with the web links to the virtual consent form and surveys on occasion. We send links via email and text and avoid certain accounts (e.g., Apple, me.com, iCloud). These limitations make clear communication, reminders, and tracking essential for staff to smoothly navigate the time between screening and consent, and consent and enrollment.

Fifth, the sheer numbers and timing of the surveys and forms for screening, consenting, data collection, and tracking provide challenges during enrollment and data collection in a longitudinal cohort study. The advantage is the comprehensive nature of the information collected over time and the ability to answer important research questions, but managing the forms (i.e., e-consent and paper consents as needed) and data in a timely way can be overwhelming. For example, we document past 30-day tobacco use on each survey and each survey link is active for a 30-day window. If the participant receives the link to complete the survey but delays past 30 days, the survey link expires. This creates the need for extra staff time to obtain a new link, contact and remind the participant to complete, and track completion of the survey.

Lastly, the built-in continuous quality improvement process using disposition and status codes allows us to track progress in real time and create feedback loops. This process provides a snapshot of the recruitment and retention landscape to inform and revise staff assignments and needed contacts. The changing numbers and types of contacts needed and limited staff availability provide regular challenges and the need to pivot staff assignments. For example, for retention, we assign one person to a 2-week block to reach all participants in need of follow-up, and then revert to the assigned field staff to follow up with specific participants during their final 2 weeks of wave eligibility. Sometimes, participants miss a survey due to our inability to reach them within the 30-day window, but we invite participants back at each wave to maintain our cohort. This continuous quality improvement and pivot process fosters participant retention.

### 4.2 Future directions

Future directions for the AppalTRUST Cohort include (a) linking the cohort survey responses to other data such as electronic health records and biomarker data, (b) broadening focus to include intervention(s) such as participation in screening and/or cessation programs, or supplementing information for other substance use or disease conditions, and (c) expanding the cohort in meaningful ways including replenishment as well as subpopulations such as youth and family dyads. We are currently conducting pilot studies in these areas of research and have plans to expand our focus to sustain the work of AppalTRUST and the continuity of the Cohort.

Immediate next steps for our AppalTRUST Center are to complete data collection for NPS and PS and publish sample characteristics and CONSORT data to demonstrate which recruitment and retention strategies are most effective. We will also examine areas of improvement to refine and enhance our protocols.

## 5 Conclusion

Our four-pronged CEnRT approach provides a flexible template for how to engage Appalachian or other underserved rural communities in public health research. While such work is both cost- and time-intensive to do well, it provides the best chance to ensure that the voices of Appalachian residents are fully represented in informing FDA CTP priorities and product regulation and provides guidance for the field on successful approaches to engaging this population.

With accurate and consistent data on the prevalence and longitudinal trends of tobacco and nicotine use and exposures among Appalachians, as well as comprehensive data on tobacco marketing and sales, the FDA could implement regulatory actions with the potential to reduce tobacco disparities in rural Appalachia and rural America at large. For example, relevant regulations related to the availability and marketing of tobacco/nicotine products - including novel products, flavors, devices, and nicotine content - could help lessen tobacco-related disparities and health-related consequences in these areas. This is especially critical in rural areas of the U.S. due to the relatively limited disincentives for use relative to their urban counterparts, including more limited enforcement of Tobacco 21 (age-of-sale) laws, fewer smoke-free laws in public places, lower tobacco prices and taxes, marketing and product availability disparities, (58) greater tobacco retailer density per capita, (59) and higher exposure to tobacco industry advertising.

AppalTRUST will contribute to TRS specifically and to public health generally by completing data collection and STROBE diagrams, providing guidance on successful recruitment and retention methods in Appalachia, analyses and publication of our findings to ensure Appalachian voices are included in rigorous research, and dissemination of results in community-facing venues.

## Data Availability

The raw data supporting the conclusions of this article will be made available by the authors, without undue reservation.

## References

[1] University of Wisconsin Population Health Institute. County Health Rankings and Roadmaps. Available online at: https://www.countyhealthrankings.org/health-data/community-conditions/health-infrastructure/health-promotion-and-harm-reduction/adult-smoking?anchor=data-methods&selected-tab=methods&year=2025 (Accessed February 11, 2026).

[2] Cornelius ME Wang TW Jamal A Loretan CG Willis G Graham-Glover B . State-specific prevalence of adult tobacco product use and cigarette smoking cessation behaviors, United States, 2018–2019. Preventing Chronic Disorder. (2023) 20:E107. doi: 10.5888/pcd20.230132 37972604 PMC10684279

[3] Kentucky Department for Public Health and the Centers for Disease Control and Prevention. Data from: Kentucky Behavioral Risk Factor Survey. (2018–2022).

[4] Higgins ST Kurti AN Palmer M Tidey JW Cepeda-Benito A Cooper MR . A review of tobacco regulatory science research on vulnerable populations. Preventive Medicine. (2019) 128:105709. doi: 10.1016/j.ypmed.2019.04.024 31054904 PMC6824984

[5] York NL Rayens MK Zhang M Jones LG Casey BR Hahn EJ. Strength of tobacco control in rural communities. J Rural Health. (2010) 26:120–8. doi: 10.1111/j.1748-0361.2010.00273.x 20446998 PMC2948793

[6] Hafez AY Gonzalez M Kulik MC Vijayaraghavan M Glantz SA. Uneven access to smoke-free laws and policies and its effect on health equity in the United States: 2000–2019. Am J Public Health. (2019) 109:1568–75. doi: 10.2105/AJPH.2019.305289 31536405 PMC6775904

[7] Hendryx M Luo J Borders T. Health disparities in Appalachia. Health Aff. (2017) 36:2213. doi: 10.1377/hlthaff.2017.1243 29200341

[8] Milliken T Beiler D Hoffman S Olenginski A Troiani V. Recruitment in Appalachian, rural and older adult populations in an artificial intelligence world: study using human-mediated follow-up. JMIR Formative Research. (2024) 8:e38189. doi: 10.2196/38189 39173153 PMC11377916

[9] Kim NH Wilson N Mashburn T Reist L Westrick SC Look K . Lessons learned recruiting a diverse sample of rural study participants during the COVID-19 pandemic. Int J Drug Policy. (2021) 97:103344. doi: 10.1016/j.drugpo.2021.103344 34186474 PMC8556070

[10] Garnett A Northwood M. Recruitment of community-based samples: experiences and recommendations for optimizing success. Can J Nurs Res. (2022) 54:101–11. doi: 10.1177/08445621211060935 34841904 PMC9109582

[11] Nichols EG Shreffler-Grant J Weinert C. Where have they gone? Recruiting and retaining older rural research participants. Online J Rural Nurs Health Care. (2021) 21:179. doi: 10.14574/ojrnhc.v21i1.642 34744525 PMC8570614

[12] Drake C Zhang Y Chaiyachati KH Polsky D. The limitations of poor broadband internet access for telemedicine use in rural America: an observational study. Ann Intern Med. (2019) 171:382–4. doi: 10.7326/M19-0283 31108509

[13] Appalachian Regional Commission. Computer and broadband access in Appalachia. ARC Chartbook. (2024). Available online at: https://www.arc.gov/about-the-appalachian-region/the-chartbook/computerand-broadband-access-in-appalachia/ (Accessed May 1, 2026).

[14] Curtis ME Clingan SE Guo H Zhu Y Mooney LJ Hser YI. Disparities in digital access among American rural and urban households and implications for telemedicine-based services. The Journal of Rural Health. (2022) 38:512–8. doi: 10.1111/jrh.12614 34355427 PMC9827725

[15] Hamby S Taylor E Mitchell K Jones L. Technology in rural Appalachia: cultural strategies of resistance and navigation. Int J Commun. (2018) 12:1248–68. https://ijoc.org/index.php/ijoc/article/view/7052

[16] Lister JJ Joudrey PJ. Rural mistrust of public health interventions in the United States: a call for taking the long view to improve adoption. Journal of Rural Health. (2022) 39:18. doi: 10.1111/jrh.12684 35691930 PMC10084067

[17] Hardy LJ Hughes A Hulen E Figueroa A Evans C Begay RC. Hiring the experts: best practices for community-engaged research. Qualitative Research. (2016) 16:592–600. doi: 10.1177/1468794115579474 27833454 PMC5100673

[18] Gibbons MM Brown EC Daniels S Rosecrance P Hardin EE Farrell I. Building on strengths while addressing barriers: career interventions in rural Appalachian communities. J Career Dev. (2019) 46:637–50. doi: 10.1177/0894845319827652 31662596 PMC6818261

[19] Sanders Thompson VL Ackermann N Bauer KL Bowen DJ Goodman MS. Strategies of community engagement in research: definitions and classifications. Transl Behav Med. (2021) 11:441–51. doi: 10.1093/tbm/ibaa042 32421173 PMC8135186

[20] Anise A. Measure What Matters: Meaningful Community Engagement. 2024: National Academy of Medicine and NAM Leadership Consortium.

[21] Tumiel-Berhalter L Brazhnik O Aguilar-Gaxiola S Brown A Carter-Edwards L Elmi A . Opportunities for facilitating community-engaged research. (Chapter 5). In:Cohn E, editor in principles of community engagement 3 ed: ATSDR. (2025).

[22] Rayens MK Kingsolver AE Christian WJ Hahn EJ Waters TM Rose SW . Considering relative rurality in tobacco regulatory science. Nicotine & Tobacco Research. (2024) 27:1147–50. doi: 10.1093/ntr/ntae303 39700459 PMC12095801

[23] Routen A Bambra C Willis A Khunti K. Hard to reach? Language matters when describing populations underserved by health and social care research. Public Health. (2022) 205:e28–9. doi: 10.1016/j.puhe.2022.02.006 35282902

[24] Mendivil-Aguayo P Rivera M Armendariz D Perez Rodriguez D Vasquez C Regino L . Zoom & whatsapp digital information and communication technologies. (ICTs) enhance community engaged research with women immigrants from Mexico. J Community Pract. (2024) 32:212–37. doi: 10.1080/10705422.2024.2351935 38883275 PMC11174975

[25] United States Department of Agriculture - Economic Research Center. Rural-Urban Commuting Area Codes. (2019). Available onlin at: https://www.ers.usda.gov/data-products/rural-urban-commuting-area-codes/ (Accessed February 11, 2026).

[26] Waldorf B Kim A. Defining And Measuring Rurality In The US: From Typologies To Continuous Indices. Commissioned paper presented at the Workshop on Rationalizing Rural Area Classifications, Washington, DC. (2015).

[27] Inagami S Gao S Karimi H Shendge MM Probst JC Stone RA. Adapting the Index of Relative Rurality. (IRR) to estimate rurality at the ZIP code level: a rural classification system in health services research. J Rural Health. (2016) 32:219–27. doi: 10.1111/jrh.12148 26397170

[28] Christian WJ Levy Jeff Rayens Mary Kay Kingsolver . Index of relative rurality. (IRR) for multiple geographies. Ann Arbor, MI: Inter-university Consortium for Political and Social Research. (2025).

[29] U.S. Census Bureau. American Community Survey 5-Year Data, 2019–2024. (2024) [updated 2/25/2024]. Available from: https://www.census.gov/data/developers/data-sets/acs-5year.html (Accessed February 11, 2026).

[30] Villarroel MA Cha AE Vahratian A. Electronic cigarette use among US adults, 2018. NCHS Data Brief. (2020) 365:1–8. 32487293

[31] Dever JA. Combining probability and nonprobability samples to form efficient hybrid estimates: an evaluation of the common support assumption. In: Proceedings of the 2018 Federal Committee on Statistical Methodology. (FCSM) Research Conference; 2018.

[32] Iachan R Berman L Kyle TM Martin KJ Deng Y Moyse DN . Weighting nonprobability and probability sample surveys in describing cancer catchment areas. Cancer Epidemiol Biomark Prev. (2019) 28:471–7. doi: 10.1158/1055-9965.EPI-18-0797 30642842 PMC6401258

[33] Wiśniowski A Sakshaug JW Perez Ruiz DA Blom AG. Integrating probability and nonprobability samples for survey inference. J Surv Stat Methodol. (2020) 8:120–47. doi: 10.1093/jssam/smz051

[34] Elliott MR Valliant R. Inference for nonprobability samples. Stat Sci. (2017): 249–64. doi: 10.1214/16-STS598

[35] Yang S Kim JK Song R. Doubly robust inference when combining probability and non-probability samples with high dimensional data. J R Stat Soc Ser B Methodol. (2020) 82:445–65. doi: 10.1111/rssb.12354 33162780 PMC7644042

[36] Melissa Data Corporation. Melissa (2026). Available online at: https://www.melissa.com/ (Accessed February 11, 2026).

[37] American Association for Public Opinion Research. Standard definitions: Final dispositions of case codes and outcome rates for surveys. American Association for Public Opinion Research. (2023).

[38] Teague S Youssef GJ Macdonald JA Sciberras E Shatte A Fuller-Tyszkiewicz M . Retention strategies in longitudinal cohort studies: a systematic review and meta-analysis. BMC Med Res Methodol. (2018) 18:151. doi: 10.1186/s12874-018-0586-7 30477443 PMC6258319

[39] Hamilton CM Strader LC Pratt JG Maiese D Hendershot T Kwok RK . The PhenX toolkit: get the most from your measures. Am J Epidemiol. (2011) 174:253–60. doi: 10.1093/aje/kwr193 21749974 PMC3141081

[40] United United States Department of Health Human Services National National Institutes of Health National National Institute on Drug Abuse Food Drug Administration . Data from: Population Assessment of Tobacco and Health. (PATH) Study. Inter-university Consortium for Political and Social Research. (2020).

[41] Centers for Disease Control and Prevention. National Youth Tobacco Survey, 2021 Questionnaire. Atlanta, Georgia: (2021).

[42] Feng J Sosnoff CS Bernert JT Blount BC Li Y Del Valle-Pinero AY . Urinary nicotine metabolites and self-reported tobacco use among adults in the Population Assessment of Tobacco and Health. (PATH) study, 2013–2014. Nicotine and Tobacco Research. (2022) 24:768–77. doi: 10.1093/ntr/ntab206 35348786 PMC9116621

[43] Tourangeau R Yan T Sun H Hyland A Stanton CA. Population assessment of tobacco and health. (PATH) reliability and validity study: aelected reliability and validity estimates. Tob Control. (2019) 28:663–8. doi: 10.1136/tobaccocontrol-2018-054561 30297373

[44] Caraballo RS Giovino GA Pechacek TF Mowery PD. Factors associated with discrepancies between self-reports on cigarette smoking and measured serum cotinine levels among persons aged 17 years or older: third national health and nutrition examination survey, 1988–1994. Am J Epidemiol. (2001) 153:807–14. doi: 10.1093/aje/153.8.807 11296155

[45] Agaku IT King BA. Validation of self-reported smokeless tobacco use by measurement of serum cotinine concentration among US adults. Am J Epidemiol. (2014) 180:749–54. doi: 10.1093/aje/kwu182 25125690 PMC4519345

[46] SAS Institute. SAS/Stat User's Guide, Version 9.4. (2017).

[47] Zou G. A modified Poisson Regression approach to prospective studies with binary data. Am J Epidemiol. (2004) 159:702–6. doi: 10.1093/aje/kwh090 15033648

[48] White IR Royston P Wood AM. Multiple imputation using chained equations: issues and guidance for practice. Stat Med. (2011) 30:377–99. doi: 10.1002/sim.4067 21225900

[49] Yuan YC. Multiple Imputation For Missing Data: Concepts And New Development. (Version 9.0). Rockville, MD: SAS Institute Inc. (2010). p. 12.

[50] Sterne JA White IR Carlin JB Spratt M Royston P Kenward MG . Multiple imputation for missing data in epidemiological and clinical research: potential and pitfalls. BMJ. (2009) 338:b2393. doi: 10.1136/bmj.b2393 19564179 PMC2714692

[51] Little RJ Rubin DB. Statistical Analysis with Missing Data. John Wiley & Sons. (2019).

[52] Cohen J. Statistical Power Analysis for the Behavioral Sciences. 2nd ed: Routledge. (1988).

[53] Von Elm E Altman DG Egger M Pocock SJ Gøtzsche PC Vandenbroucke JP. The strengthening the reporting of observational studies in epidemiology. (STROBE) statement: guidelines for reporting observational studies. Lancet. (2007) 370:1453–7. doi: 10.1016/S0140-6736(07)61602-X 18064739

[54] Kegler MC Hermstad A Smith AJ Bigger L Smith TA Line MG . Community readiness to address health equity: progress and challenges across eleven Georgia communities. SSM - Qual Res Health. (2026) 9:100777. doi: 10.1016/j.ssmqr.2026.100777

[55] Savla J Pelletier T Roberto KA DiFeliceantonio AG Fullen M Hosig K . Whole health, whole communities—Dialogues to reduce rural health disparities. NEJM Catal Innov Care Deliv. (2026) 7:1–10. doi: 10.1056/CAT.26.0094 42455988

[56] Carroll D McClernon FJ Frisbee S Bittencourt L Rubenstein D Gwon J . Tobacco product regulation: opportunities for advancing health equity in rural America. Nicotine and Tobacco Research. (2025) 27:2333–7. doi: 10.1093/ntr/ntaf104 40454948

[57] U.S. Census Bureau. County-to-County Migration Flows: 2016-2020 American County Survey. (2023). Available online at: https://www.census.gov/topics/population/migration/guidance/county-to-county-migration-flows.html (Accessed May 1, 2026).

[58] Feizy S van de Venne J Abadi M Christian WJ Jayawardhana J Fennell BS . Commercial tobacco retail marketing in rural communities in Appalachian Kentucky. Preventive Medicine Reports. (2024). (2025):103354. doi: 10.1016/j.pmedr.2025.103354 42460280 PMC13370820

[59] Rose SW Christian WJ Venne Jvd Mattingly D Fennell BS Feizy S . The interaction of tobacco retailer density and rurality with tobacco use prevalence in Kentucky. J Rural Health. (2026) 42:e70106. doi: 10.1111/jrh.70106 41454484 PMC12884400

[60] Piper ME Piasecki TM Federman EB Bolt DM Smith SS Fiore MC . A multiple motives approach to tobacco dependence: the wisconsin inventory of smoking dependence motives. (WISDM-68). J Consult Clin Psychol. (2004) 72:139. doi: 10.1037/0022-006X.72.2.139 15065950

[61] Shiffman S Waters AJ Hickcox M. The nicotine dependence syndrome scale: a multidimensional measure of nicotine dependence. Nicotine & Tobacco Research. (2004) 6:327–48. doi: 10.1080/1462220042000202481 15203807

[62] American Psychiatric Association. Diagnostic and statistical manual of mental disorders (DSM-5^Ⓡ^), 5th ed. Arlington, VA: American Psychiatric Publishing (2013). doi: 10.1176/appi.books.9780890425596

[63] Strong DR Pearson J Ehlke S Kirchner T Abrams D Taylor K . Indicators of dependence for different types of tobacco product users: descriptive findings from Wave 1. (2013–2014) of the Population Assessment of Tobacco and Health. (PATH) study. Drug Alcohol Depend. (2017) 178:257–66. doi: 10.1016/j.drugalcdep.2017.05.010 28675817

[64] Choi WS Gilpin EA Farkas AJ Pierce JP. Determining the probability of future smoking among adolescents. Addiction. (2001) 96:313–23. doi: 10.1046/j.1360-0443.2001.96231315.x 11182877

